# Temporal resource continuity for egg parasitoids of *Dalbulus maidis* (Hemiptera: Cicadellidae) during winter on irrigated maize crops and edge grasses

**DOI:** 10.1093/jisesa/ieae079

**Published:** 2024-08-16

**Authors:** Gustavo Moya-Raygoza

**Affiliations:** Departamento de Botánica y Zoología, CUCBA, Universidad de Guadalajara, Zapopan, Jalisco, Mexico

**Keywords:** Hymenoptera, biological control, agroecosystem, insect ecology

## Abstract

Little is known about winter-season parasitism of eggs of the corn leafhopper *Dalbulus maidis* DeLong (Hemiptera: Cicadellidae), an important pest of maize throughout the Americas. Our study, conducted in Mexico, aimed to characterize winter-season parasitism of corn leafhopper eggs on maize crops cultivated with drip irrigation and on wild grasses that grow on the edges of maize crops when maize is not present. Maize leaves baited with *D*. *maidis* eggs were used to trap the egg parasitoids in the field. In the first year (2022), parasitism of *D. maidis* eggs was investigated in maize fields planted contiguously on different dates (asynchronous planting). In the second year (2023), parasitism of *D. maidis* eggs was evaluated in edge grasses and in adjacent maize crops planted on the same date (synchronous). The highest percentage of parasitism (53%), percentage of emergence, and total abundance of egg parasitoids were found in asynchronous maize fields. Here, *Anagrus virlai* Triapitsyn (Hymenoptera: Mymaridae), *Paracentrobia subflava* (Girault) (Hymenoptera: Trichogrammatidae), and *Pseudoligosita* sp. (Hymenoptera: Trichogrammatidae) wasps were found parasitizing the *D. maidis* eggs, with *P. subflava* being the most abundant. In wild edge grasses, only *P. subflava* was found, showing low levels of parasitism, while in synchronous maize, *P. subflava* increased its percentage of parasitism (up to 37%), percentage of emergence, and abundance, during winter. These results suggest that *P. subflava* acts as an efficient biological control agent of *D. maidis* in irrigation-grown maize crops during the winter season, and that edge grasses are overwinter habitats for *P. subflava.*

## Graphical Abstract

**Graphical Abstract:**
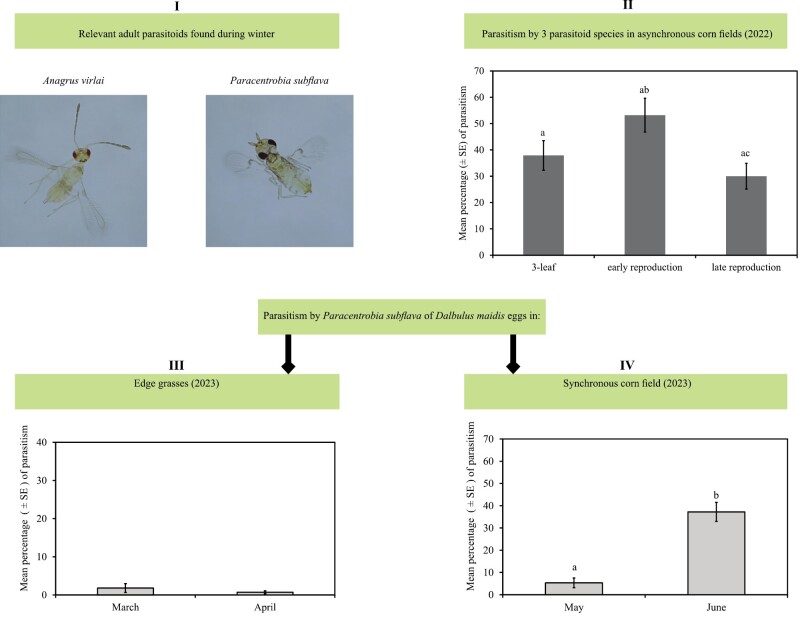

## Introduction

Maize (*Zea mays* ssp*. mays* L.; Poales: Poaceae) is one of the most important crops, in terms of production for direct and indirect human consumption, in the world (Ranum et al. 2014). On the American continent, the United States and Brazil are the highest producers of corn (Erenstein et al. 2022) and the highest consumers of water for corn production (Foley et al. 2020). Climate change could cause challenges to corn production, affecting both rainfed and irrigated agriculture in the coming years (Foley et al. 2020). In Mexico, the maize yield is predicted to be affected by future climatic change in rainfed and irrigated crop areas (Ureta et al. 2020). In Mexico, 81.25% of the corn is cultivated with rain during the wet season and 18.75% of the corn is cultivated with irrigation during the dry season, which is the winter season (Murray-Tortarolo et al. 2018). Linear models predict that climate change will affect maize production in Mexico (Ureta et al. 2020). These authors predict higher yield stability for irrigated maize fields than for rainfed maize fields, and corn yields from rainfed fields will be reduced under future scenarios.

In the United States, maize yield increases by 55% when using drip irrigation during the dry season vs. using furrow irrigation (Lamm and Trooien 2003). In recent years, drip irrigation has been widely applied to maize production by farmers in American countries like the United States, Brazil, and Argentina, and this has saved water in irrigated agriculture (Praveen Rao et al. 2021). On the other hand, insect pests have not been extensively investigated in maize cultivated with drip irrigation. Little is known about the natural enemies of the insect pests in winter habitats free of maize, and in irrigated maize during the winter season.

The corn leafhopper *Dalbulus maidis* (DeLong, 1923) (Hemiptera: Cicadellidae) is one of the most important pests of maize throughout the American continent (Nault 1990). Nymphs and adults of *D. maidis* are specialists on maize, and transmit plant pathogens, including corn stunt spiroplasma, maize bushy stunt phytoplasma, and maize rayado fino virus (Nault 1980). During the winter season, the corn leafhopper is abundant in maize cultivated with irrigation in Mexico (Moya-Raygoza et al. 2007), and parasitoids, including Diptera, Hymenoptera, and Strepsiptera, parasitize nymphs and adults of the corn leafhopper in these irrigated maize fields (Moya-Raygoza et al. 2004). In Mexico, Moya-Raygoza and Becerra-Chiron (2014) found that eggs of the corn leafhopper are parasitized by the wasp *Oligosita* sp. (Hymenoptera: Trichogrammatidae) during the winter season on perennial grasses, volunteer maize, and irrigated maize habitats; however, the authors did not report parasitism traits such as percentage of parasitism and percentage of emergence. In the United States (California), *D. maidis* is abundant throughout the winter on volunteer corn and on cultivated corn in April and May (Summers et al. 2004), but little is known about the parasitoids of *D. maidis* in the United States. In Brazil, a high number of *D. maidis* adults have been found throughout the winter season on maize cultivated with irrigation (Oliveira et al. 2013). However, no study has been performed in Brazil to investigate the parasitoids of *D. maidis* during the winter season on maize cultivated with irrigation.

Egg parasitoids are efficient biological control agents against insect pests (Mills 2010, Sann et al. 2018). However, little is known about the egg parasitoids that attack the corn leafhopper in the different habitats throughout the winter season. In the winter season, once the maize crop is harvested, there are wild green perennial grasses on the edges. At the end of the winter season, farmers grow maize using irrigation. Green perennial edge grasses harbor sap-feeding non-*D. maidis* leafhoppers that can be used by egg parasitoids as alternative hosts, and maize planted throughout the winter season, cultivated with drip irrigation, could also harbor egg parasitoids of *D. maidis*. If this agroecosystem is cultivated, when farmers begin planting maize during the wet season, which is the main maize-growing season, egg parasitoids of the corn leafhopper will already be present and capable of quickly spreading from corn cultivated in the winter season to corn cultivated during the maize-growing wet season within the same region.

In the first year, the aim of the present 2-yr study was to investigate the parasitism traits of the egg parasitoids that attack *D. maidis* in maize planted in adjacent fields at different dates (asynchronous) throughout the winter season using drip irrigation. In the second year, the aim was to investigate the parasitism traits of the egg parasitoids of *D. maidis* on wild grasses that grow on the edges and on adjacent maize crops cultivated with drip irrigation during the winter season. Furthermore, the abundance of leafhopper adults on wild edge grasses and on maize crops in this second year was investigated. This is the first study that investigates parasitoids of an important insect pest on maize crops cultivated with drip irrigation, a method that saves water and increases corn yield. Knowing the egg parasitoid species and their levels of parasitism in the different habitats during the winter will contribute to the management of corn leafhopper populations.

## Materials and Methods

The experiments were performed during the winter season in Zapopan region, in the west-central state of Jalisco, Mexico, to research the egg parasitoids of *D. maidis*. The experimental site was located at 20° 44ʹ 40.6″ N, 103° 30ʹ 57.7″ W, 1,662 m elevation. The first experiment was performed at the end of the winter season in July 2022 over 3 maize fields planted adjacent to each other on different dates (asynchronous) and grown with drip irrigation. The second experimental series was conducted in March and April of 2023, looking at parasitoids gathered from green wild grasses from the edges of maize fields when the maize crop was absent, and in May and June of 2023, looking at parasitoids gathered from adjacent maize fields planted at the same time and cultivated with drip irrigation.

### Collecting the Egg Parasitoids

Baited maize plants (sentinel plants), which were grown from maize seeds of the landrace “maiz ancho,” were used to sample egg parasitoids of the corn leafhopper during both sets of field experiments (2022 and 2023). These were potted (size of the pot 15.0 × 10.0 cm) maize plants with *D. maidis* eggs that were exposed to the community of egg parasitoids present in each experimental field.

To sample egg parasitoids, *D. maidis* adults were first collected from the Zapopan region and reared in the greenhouse. The temperatures in the greenhouse were between 18 and 30 °C, and adults were reared in cages (size 45.0 × 40.0 × 30.0 cm) using the landrace “maiz ancho,” which received water every day. These reared *D. maidis* adults were used in all the experiments. For oviposition of *D. maidis* females, leaf cages (size 4.0 × 5.5 × 2.0 cm, with a small hole covered with fine mesh) were attached to a live, potted maize plant such that each cage enclosed a single leaf. Seven *D. maidis* females, 2 wk old, were placed in each single-leaf cage on the landrace maize known as “maiz ancho.” Each potted maize plant was at the 5-leaf stage (when the plant has 5 leaves developed) at the moment of *D*. *maidis* oviposition. Female *D. maidis* were allowed to oviposit in a rearing room at 25 ± 2 °C, 50% relative humidity, and a photoperiod of 12:12 (L:D) h for 72 h. After 72 h, the *D. maidis* females were removed, and the pots containing the leaves with eggs (sentinel plants) were transported to the experimental fields at Zapopan. The number of eggs deposited by *D. maidis* females on sentinel plants was not counted because eggs are difficult to observe during the first hours once oviposited. Pots containing sentinel landrace maize were placed in each experimental field. Each pot contained 1 live maize landrace baited with *D. maidis* eggs. The pots received water if required and remained in the maize field for 5 d to allow exposure to egg parasitoids. After 5 d, the plants with sentinel eggs were returned to the laboratory.

Once returned to the laboratory, pots were maintained in the rearing room, and 3 or 4 d later, the total number of parasitized and non-parasitized *D. maidis* eggs on each exposed leaf was counted using a stereomicroscope (Stemi DV4; Carl Zeiss, Germany). Parasitized eggs are visually identifiable as they show a red or black color a few days after being parasitized. Once the eggs were counted, the leaves with eggs were cut from the maize plant and transferred to a Petri dish (size 90 × 18 mm) with wet tissue paper. The tissue paper was replaced when required to avoid fungus development. Each dish, each with 1 leaf, was covered with clear plastic food wrap to prevent the escape of emerging adult parasitoids and was maintained in the rearing room under the conditions described above. The Petri dishes were checked every other day for 35 d to collect all emerging adult parasitoids, which were deposited in 95% ethanol for future identification. All adult parasitoids that emerged were counted and identified using the associated morphological keys of Pinto (2006) and Triapitsyn et al. (2019). Representatives of each morphospecies were slide-mounted in Faure media. For each sentinel maize leaf, the number of *D. maidis* oviposited, the number of eggs with evidence of parasitism (eggs with a black or red color), the number of parasitic wasps that emerged, and the species that emerged were determined. To obtain the percentage of parasitism, the total number of eggs with coloration from each maize leaf was divided by the number of eggs laid on each leaf, multiplied by 100. Also, to obtain the percentage of emergence, the total number of adult parasitoids that emerged from each maize leaf was divided by the number of eggs laid on each leaf, multiplied by 100.

### Egg Parasitoids on Asynchronous Maize

The first experiment was performed in 3 adjacent maize fields (treatments) that had been planted on different dates (March, May, and June) but were all sampled in July. Maize plants in the field were, therefore, at different stages when the pots with the egg-baited maize leaves were set in the field; those planted in March had mature ears, those planted in May had young ears, and those planted in June were at the 3-leaf stage. The treatment planted at the end of March was called “late reproductive stage”; the treatment planted at the end of May was called “early reproductive stage”; and the treatment planted in the first week of June was called “3-leaf stage.” In July, the pots with baited maize leaves were placed in the 3 treatments mentioned: “3-leaf stage,” “early reproductive stage,” and “late reproductive stage.” The pots were placed about 2–3 m from the edge of the maize crop along a transect, with a distance of 5 m between each pot. This was performed 3 different times per treatment. The first sampling was conducted on 4 July, the second on 11 July, and the third on 18 July; and the sampling procedure was performed 35 times (35 leaves), 34 times (34 leaves), and 37 times (37 leaves), respectively, per treatment.

### Egg Parasitoids on Edge Grasses and on Synchronous Maize

In the second experiment, egg parasitoids of *D. maidis* were sampled using pots with sentinel maize leaves on green grasses that grow naturally at the edges of maize fields. On these edge grasses, pots were placed along a transect with a distance of 5 m between each pot. In total, the procedure was performed 66 times (66 leaves) on 21 March and another 66 times (66 leaves) on 10 April.

In addition, pots with maize leaves baited with *D*. *maidis* eggs were used to trap the egg parasitoids in a field of maize that had all been planted on a single date. The pots were placed about 2–3 m from the edge of the maize crop along a transect, with a distance of 5 m between each pot. On 20 May, the procedure was carried out 53 times (53 leaves); the maize crop at this time was at the 6-leaf stage. The same procedure was performed on 26 June, was performed 57 times (57 leaves), and the maize crop at this time was in the reproductive stage.

### Leafhopper Adults

Leafhopper adults were surveyed during the winter season of 2023 on the edge grasses and on maize crops in the experimental site, where baited maize plants were set. Specifically, on 15 March, leafhoppers were sampled from the green grasses that grow in the maize edges, to determine the presence of leafhopper adults, including *D. maidis* adults. A total of 600 sweeps were performed in 3 different transects over the green grasses. In addition, on 29 May, a total of 600 sweeps were conducted in 3 different transects to collect *D*. *maidis* adults over the foliage of maize plants.

### Statistical Analysis

The number of *D. maidis* eggs, the number of adult parasitoids that emerged from the eggs, the percentage of parasitism, and the percentage of emergence among treatments and in each experiment were compared using the generalized linear model (family = Poisson). For all pairwise comparisons, the estimated marginal means were calculated. Statistical analysis was performed with SPSS version 22 (IBM corporation 2013).

## Results

### Egg Parasitoids on Asynchronous Maize

The maize sentinel plants had a similar (Wald chi-square test: χ^2^ = 1.61, df = 2, *P* = 0.44) number of *D. maidis* parasitoid-attractant eggs. In the “3-leaf stage” treatment, the mean number of eggs was 47.28 (SE = 6.07); in the “early reproductive stage” treatment, the mean number of eggs was 46.08 (SE = 4.84); and in the “late reproductive stage” treatment, the mean number of eggs was 55.00 (SE = 5.24).

**Fig. 1.**
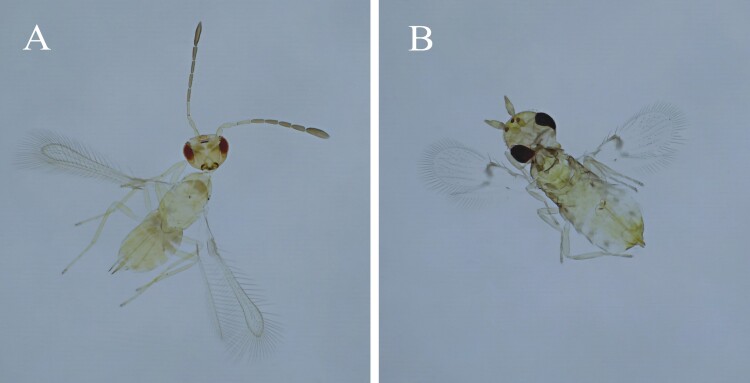
Relevant adult parasitoids emerged from *Dalbulus maidis* eggs. A) *Anagrus virlai* female. B) *Paracentrobia subflava* female.

**Fig. 2.**
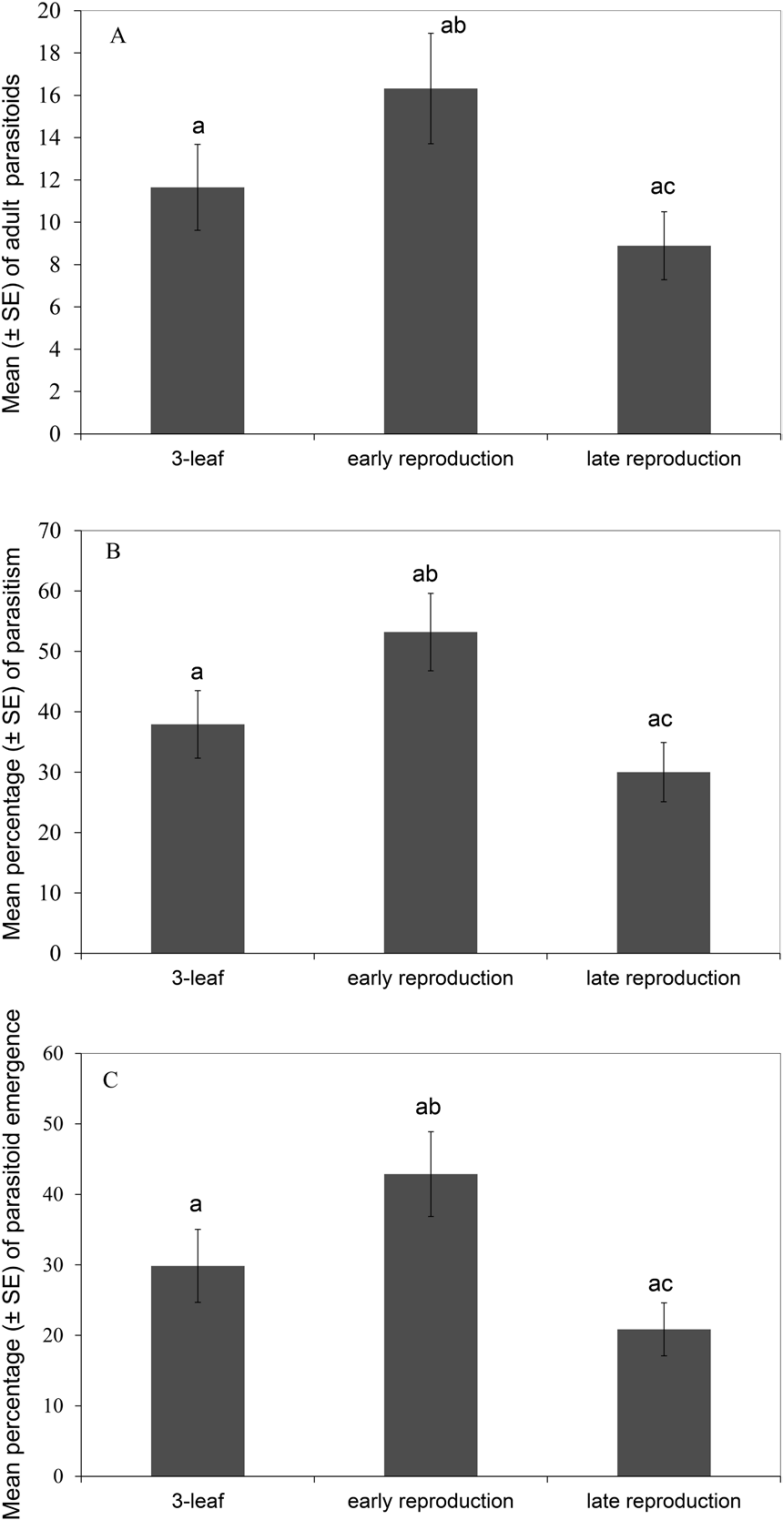
Parasitism of *Dalbulus maidis* eggs in asynchronous corn fields at the end of the winter season in July 2022, in 3 treatments. Lowercase letters denote statistical significance in mean among treatments: “3-leaf stage,” “early reproductive stage,” and “late reproductive stage.” A) Mean adult parasitoids. B) Mean percentage of parasitism. C) Mean parasitoid emergence.

*Anagrus virlai* Triapitsyn (Hymenoptera: Mymaridae) (Fig. 1A), *Paracentrobia subflava* (Girault) (Hymenoptera: Trichogrammatidae) (Fig. 1B), and *Pseudoligosita* sp. (Hymenoptera: Trichogrammatidae) were the adult solitary parasitoids that emerged from sentinel maize plants. The total abundance of parasitoids differed significantly between treatments (Wald chi-square test: χ^2^ = 6.69, df = 2, *P* = 0.03). The highest abundance of total adult parasitoids was found in the “early reproductive stage” treatment (Fig. 2A). The total percentage of parasitism differed among the 3 treatments (Wald chi-square test: χ^2^ = 7.30, df = 2, *P* = 0.02). The highest percentage of parasitism, 53%, was found in the “early reproductive stage,” treatment (Fig. 2B). In addition, this treatment also resulted in the highest percentage of parasitoids emerged: 42% (Fig. 2C).

**Fig. 3.**
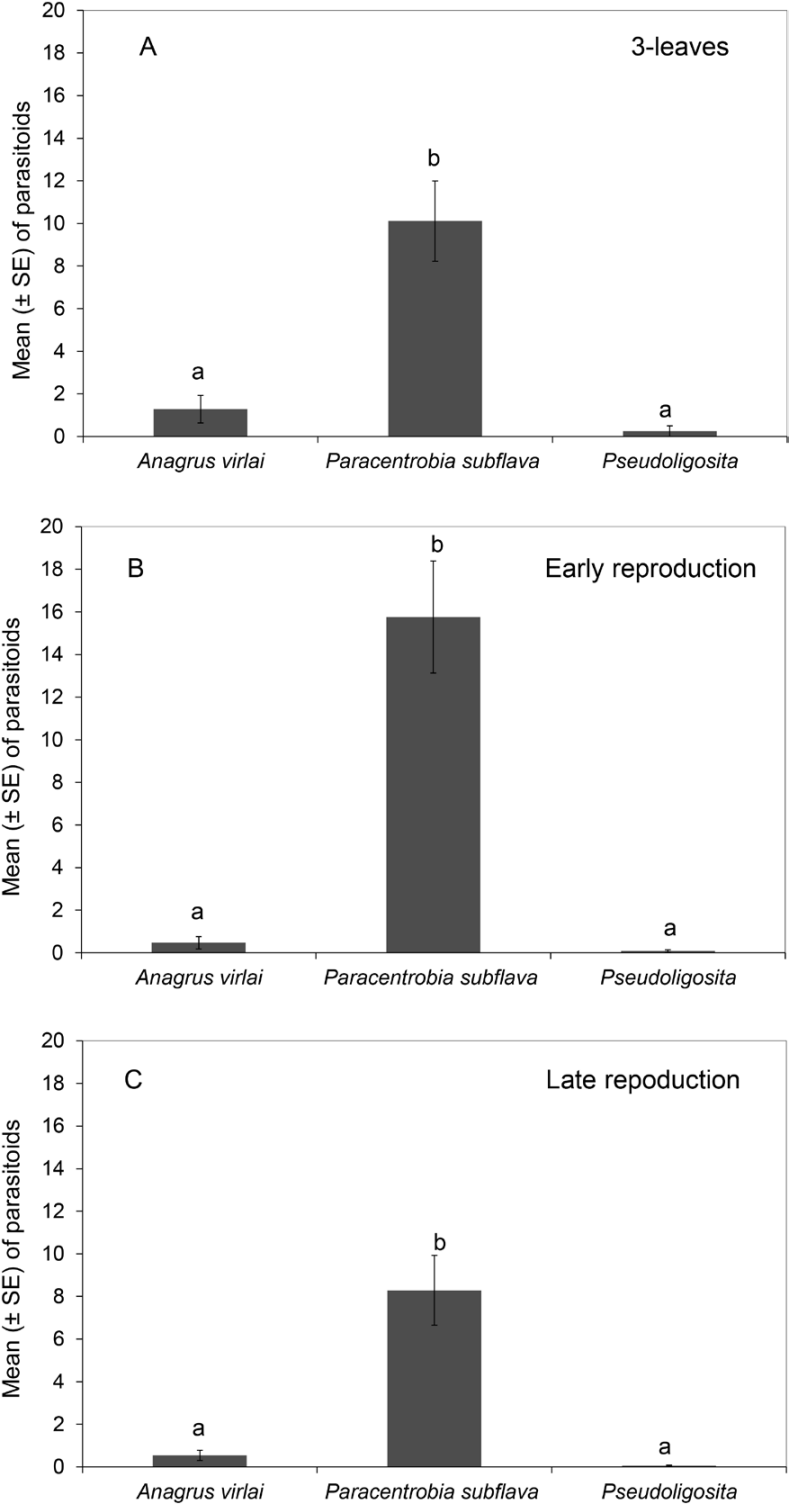
Abundance of *Anagrus virlai*, *Paracentrobia subflava*, and *Pseudoligosita* sp. emerged from *Dalbulus maidis* eggs in asynchronous corn fields at the end of the winter season (2022). Lowercase letters denote statistical significance in mean among species. A) Mean adult parasitoids in the “3-leaf stage” treatment. B) Mean adult parasitoids in the “early reproductive stage” treatment. C) Mean adult parasitoids in the “late reproductive stage” treatment.

*Paracentrobia subflava* was the most abundant parasitoid found in the treatments: “3-leaf stage” (Fig. 3A), “early reproductive stage” (Fig. 3B), and “late reproductive stage” (Fig. 3C).

### Egg Parasitoids on Edge Grasses and on Synchronous Maize

#### Edge Grasses

The maize bait plants placed in the green grasses grown at the maize edges had similar (Wald chi-square test: χ^2^ = 3.17, df = 1, *P* = 0.07) numbers of *D. maidis* eggs. In March, the mean number of eggs was 25.65 (SE = 2.99), and in April, the mean number of eggs was 20.09 (SE = 1.75).

**Fig. 4.**
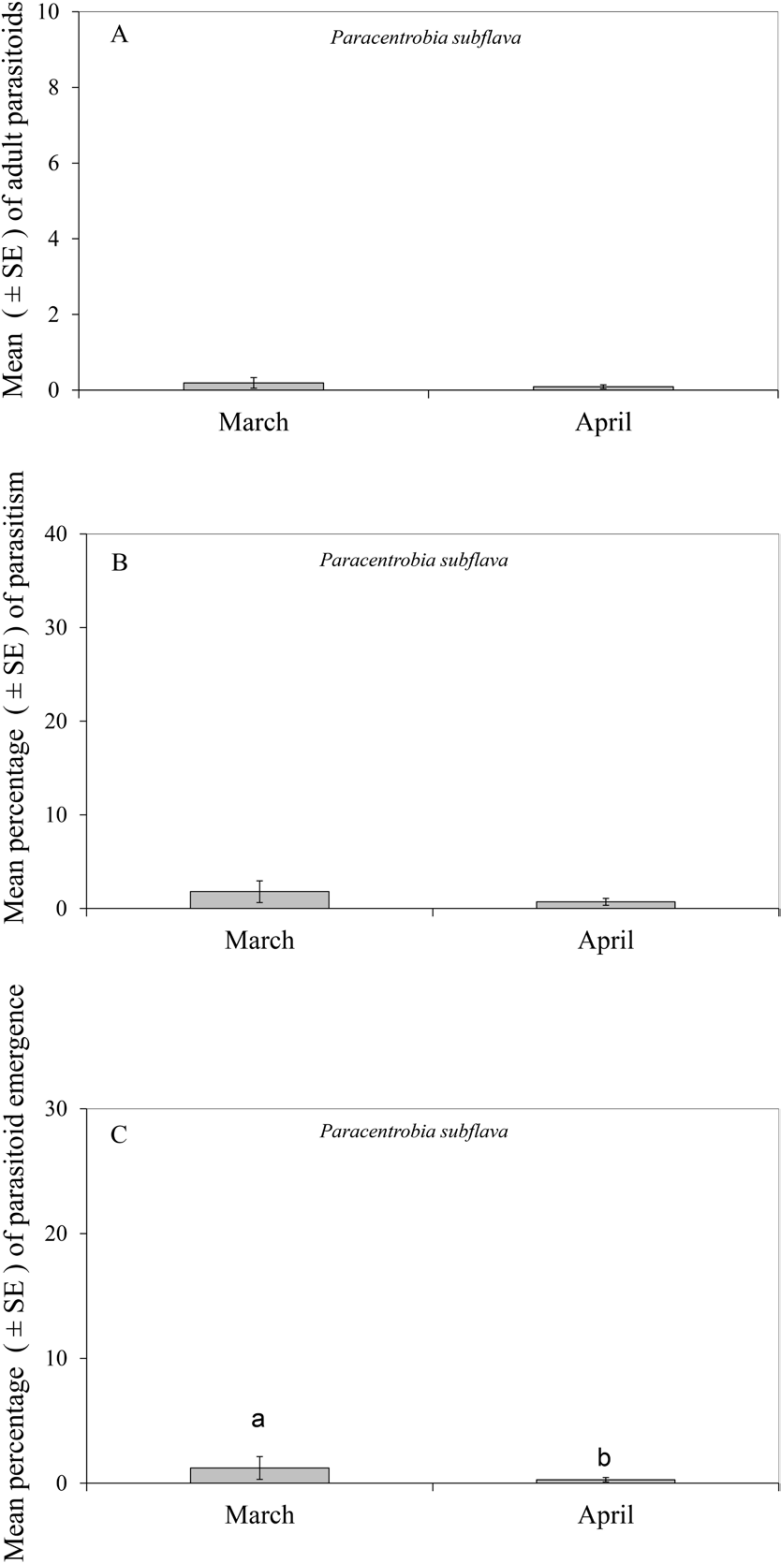
Parasitism by *Paracentrobia subflava* of *Dalbulus maidis* eggs in green edge grasses in March and April during the winter season of 2023. Lowercase letters denote statistical significance in mean among months. A) Mean adult parasitoids. B) Mean percentage of parasitism. C) Mean parasitoid emergence.

*Paracentrobia subflava* was the only parasitoid found attacking the eggs of *D*. *maidis* in the green edge grasses. The abundance of *P. subflava* adults was similar in March and April (Wald chi-square test: χ^2^ = 2.43, df = 1, *P* = 0.11), with a very low number of *P. subflava* adults in these 2 mo (Fig. 4A). In addition, no differences were found in the percentage of parasitism by *P*. *subflava* (Wald chi-square test: χ^2^ = 3.65, df = 1, *P* = 0.056), with a very low percentage of parasitism in both March and April, reaching less than 2.0% parasitism of the *D. maidis* sentinel eggs (Fig. 4B). However, the percentage of emergence of *P. subflava* (Wald chi-square test: χ^2^ = 6.02, df = 1, *P* = 0.01) did differ significantly between March and April, although both months showed less than 2.0% emergence (Fig. 4C).

#### Maize Crop

In the maize bait plants placed in the maize crop, the number of *D. maidis* eggs on the sentinel plants did differ significantly (Wald chi-square test: χ^2^ = 33. 26, df = 1, *P* = 0.0001). In May, the mean number of eggs was 24.24 (SE = 2.47) per sentinel plant, while in June, the mean number of eggs per plant was 55.91 (SE = 5.13).

**Fig. 5.**
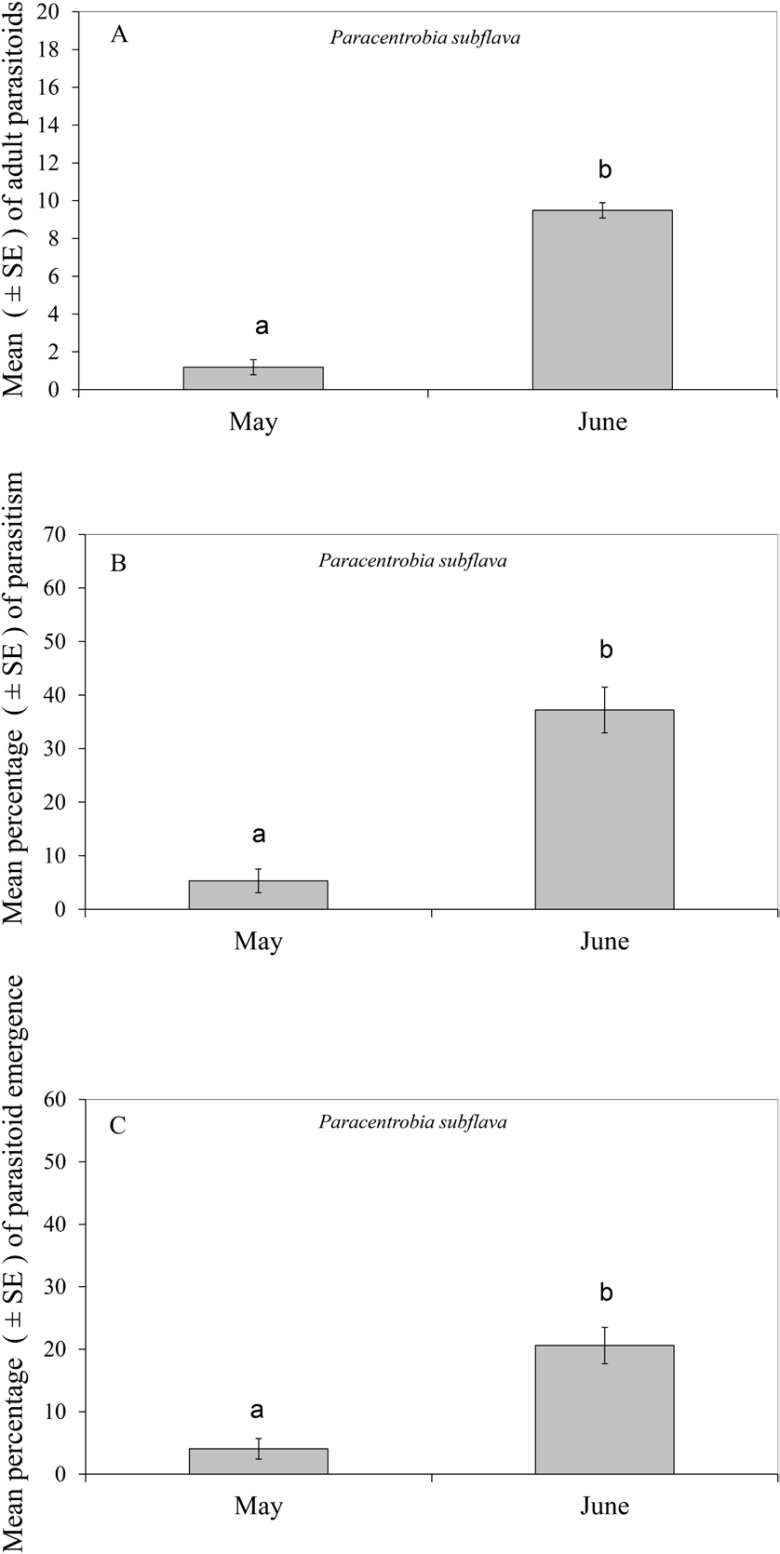
Parasitism by *Paracentrobia subflava* of *Dalbulus maidis* eggs within the maize field in May and June during the winter season of 2023. Lowercase letters denote statistical significance in mean among months. A) Mean adult parasitoids. B) Mean percentage of parasitism. C) Mean parasitoid emergence.

*Paracentrobia subflava* was the only parasitoid found attacking the *D*. *maidis* eggs placed in the maize fields planted in May and June, at the end of the winter season. The abundance of *P. subflava* adults was different in May and June (Wald chi-square test: χ^2^ = 31.77, df = 1, *P* = 0.0001), with the highest number of parasitoid adults seen in June (Fig. 5A). Also, a significant difference in the percentage of parasitism by *P*. *subflava* was found between the May and June treatments (Wald chi-square test: χ^2^ = 33.45, df = 1, *P* = 0.0001), reaching the highest percentage in June with a mean average of 37% (Fig. 5B). In addition, the percentage of emergence by *P. subflava* also differed significantly between the 2 mo (Wald chi-square test: χ^2^ = 23.28, df = 1, *P *= 0.0001), reaching the highest percentage in June, with a mean of 21% (Fig. 5C).

### Leafhopper Adults

Several species of leafhopper adults were collected on the green edge grasses on 15 March 2023. The most abundant species were *Graminella sonora* (Ball) and *Stirellus bicolor* (Van Duzee), followed by *Exitianus picatus* Gibson and *Amblysellus necopinus* DeLong and Hamilton. No *D. maidis* adults were found in the green edge grasses on 15 March 2023. However, in the maize crop, a total of 153 *D. maidis* adults were collected from the maize foliage on 29 May, when maize plants were at the 9-leaf stage.

## Discussion

Within the American continents, maize reaches the highest yields in the United States, Brazil, and Mexico (Ranum et al. 2014). In Mexico, although the corn planted with irrigation during the winter season represents only 18.75% of the maize cultivated (Murray-Tortarolo et al. 2018), future scenarios, in light of climatic change and precipitation problems, predict higher maize yield stability for irrigated maize fields than for rainfed maize fields (Ureta et al. 2020). In the American continents, *D. maidis* adults are commonly seen on maize cultivated in the winter season in countries such as the United States (California) (Summers et al. 2004), Mexico (Moya-Raygoza et al. 2007), and Brazil (Oliveira et al. 2013). In the present study, corn leafhopper adults were found in maize fields cultivated with drip irrigation, confirming the presence of *D. maidis* adults in corn cultivated during the winter season.

In the present study, several parasitism traits of parasitoids that attack the eggs of *D. maidis* were, for the first time, reported in corn cultivated during the dry (drip irrigation) season. These parasitoids were sampled from corn planted on different dates, in 2022, and from corn planted on a single date, in 2023. High total abundance and total percentage of parasitism by egg parasitoids were seen in maize planted asynchronously in the dry season, before maize fields were planted during the wet season (2022 experiments). Similar results were found with hopper parasitoids in a rice crop, another globally important cereal; asynchronous planting cycles in rice systems serve to maintain or enhance egg parasitoid populations and their biological control ability (Sann et al. 2018). In the present study, *A. virlai*, *P. subflava*, and *Pseudoligosita* sp. parasitized the eggs of *D. maidis*; however, *P. subflava* was the most abundant in the 3 tested maize treatments. The 3 egg parasitoid species together show a high percentage of parasitism of the *D. maidis* eggs in the maize field. The percentage of parasitism was highest in the “early reproductive stage” maize field treatment, reaching 53% parasitism. In contrast, in corn planted on a single date in 2023 during the dry season, the parasitism rate only reached 37%, and only *P. subflava* was found. The results from these 2 yr suggest that *P. subflava* is an efficient biological control agent, reaching a high percentage of parasitism under field conditions of winter maize cultivation with irrigation.

One advantage of using this parasitoid as a biological control agent is that artificial diets for *P. subflava* have been tested in laboratory conditions. Recent laboratory studies have found that the longevity of *P. subflava* adults was higher when fed on a honey diet than when fed on honey + pollen or honeydew diets (Torres-Moreno and Moya-Raygoza 2024).

A further advantage is that *P. subflava* developed in maize cultivated during the winter season is capable of dispersing locally to maize planted during the wet season, when most maize is cultivated. *Paracentrobia subflava* disperses in crops through small jumps and short flights. If a *P. subflava* population were already present in the winter, adults could easily move to new maize planted during the wet season and already be present in substantial numbers when *D. maidis* leafhoppers first arrive in the maize field. *Paracentrobia subflava* is a native parasitoid that attacks the eggs of *D. maidis* in the natural habitats of the annual teosinte *Z. mays* ssp. *parviglumis* Iltis & Doebley in Mexico (Moya-Raygoza and Triapitsyn 2017). This annual teosinte is the direct ancestor of maize (*Z. mays* ssp. *mays* L.) (Matsuoka et al. 2002). *Paracentrobia subflava* is broadly distributed in Latin America in corn crops cultivated in the wet season in Mexico (Torres-Moreno and Moya-Raygoza 2020, 2021), and South America (Luft Albarracin et al. 2017).

Most studies on egg parasitoids of *D. maidis* have been performed during the maize-growing wet season. During this wet season, at least 2 generations of *D. maidis* develop, and several wasp species parasitize the eggs of *D. maidis* (Moya-Raygoza et al. 2012). Of these, *P. subflava* and *A*. *virlai* are the most common parasitoids that parasitize the eggs of *D. maidis* in the maize-growing wet season (Moya-Raygoza 2019). In this wet season, the mean percentage of parasitism of *D. maidis* eggs by these 2 most abundant parasitoids reached about 22% in August and 35% in September, in the Zapopan region of Mexico (Moya-Raygoza et al. 2014).

Green edge grasses that grow during the winter season are a habitat for egg parasitoids of *D. maidis* when maize is not present. In this study, the presence of *D. maidis* adults was not found, but other leafhoppers, such as *G. sonora* and *S. bicolor*, followed by *E. picatus* and *Am. necopinus*, were collected. The leafhoppers that inhabit these edge grasses during winter are negative for corn stunt spiroplasma and maize bushy stunt phytoplasma and do not harbor these bacteria (Torres-Moreno et al. 2015). Therefore, the eggs of these leafhoppers could serve as healthy alternative hosts for *D. maidis* egg parasitoids when maize is absent.

In the present study, eggs of *D. maidis* were parasitized in March and April by *P. subflava*, although a very low percentage of parasitism was found. Similar results were reported by Moya-Raygoza and Becerra-Chiron (2014), who found that eggs of *D. maidis* were parasitized by *Oligosita* sp. in January on green edge grasses, although the percentage of parasitism was not evaluated. The low level of parasitism by egg parasitoids that attack hoppers also has been reported in rice habitats when monoculture rice is not present (Sann et al. 2018). Grassy edges provide shelter and alternative hosts for natural enemies, thereby facilitating colonization of crop fields (Holland et al. 2016, Gurr et al. 2017, Gontijo 2019). In the winter, maize field edges grow perennial grasses where adult egg parasitoids obtain food resources from the honeydew produced by non-*D. maidis* leafhoppers, such as *S. bicolor* and *G. sonora*, which are common and can be used as alternative hosts by egg parasitoids (Moya-Raygoza and Becerra-Chiron 2014). Therefore, it is important to conserve field edges with alternative, non-*D. maidis* leafhopper hosts. These leafhopper species could maintain *P. subflava* on the edges until maize is cultivated in the field with drip irrigation, ready to parasitize the eggs of *D. maidis*. Edge grasses with alternative leafhopper hosts and maize planted asynchronously with drip irrigation contribute to increasing the level of parasitism by *P. subflava* during the winter season. This can provide continuity of biological control in the maize cultivated during the wet season. Temporal resource continuity is one of the most important factors in increasing natural enemies and maintaining pest control in agroecosystems (Iuliano and Grotton 2020).

In conclusion, the parasitoids *A. virlai*, *P. subflava*, and *Pseudoligosita* sp. were found in July, before beginning the maize-growing wet season, on maize planted at different dates during the winter season using drip irrigation. In this asynchronous maize, the highest percentages of parasitism among the 3 egg parasitoids collected was 53%, by *P. subflava,* the species that reached the highest percentages of parasitism and the highest abundance. In a second year, looking at maize planted on a single date, the highest percentage of parasitism was 37%. In this condition, only *P. subflava* was found attacking the eggs of *D. maidis*. Edge grasses host leafhopper adults that are used as alternative hosts by *P. subflava*. Therefore, this parasitoid species could be used as a biological control agent against *D. maidis* in maize crops cultivated during winter. The continuity of temporal resources during the winter season contributes to the conservation and continuity of biological control.
